# The Importance of Serum Perilipin-2 Level as an Early Indicator of Inflammation in Non-Alcoholic Fatty Liver Disease

**DOI:** 10.3390/diagnostics16010106

**Published:** 2025-12-28

**Authors:** Omer Vehbi Alpaydin, Eda Nur Duran, Ayse Hur Alpaydin, Iskender Ekinci, Seyma Dumur, Hafize Uzun, Murat Akarsu, Isa Yalcinkaya, Omur Tabak

**Affiliations:** 1 Department of Internal Medicine, Kanuni Sultan Suleyman Training and Research Hospital, University of Health Sciences, Istanbul 34255, Türkiye; vehbialpaydin@gmail.com (O.V.A.); enurduran@gmail.com (E.N.D.); aysehur.93.a1@gmail.com (A.H.A.); muratakarsu79@gmail.com (M.A.); 2 Department of Internal Medicine, BezmialemVakif University, Istanbul 34093, Türkiye; driskenderekinci@gmail.com; 3 Department of Medical Biochemistry, Faculty of Medicine, Istanbul Atlas University, Istanbul 34403, Türkiye; seyma.dumur@atlas.edu.tr (S.D.); hafize.uzun@atlas.edu.tr (H.U.); 4 Department of Hematology, Basaksehir Cam and Sakura City Hospital, University of Health Sciences, Istanbul 34255, Türkiye; isayk@hotmail.com

**Keywords:** hepatic steatosis index, inflammation, non-alcoholic fatty liver disease, perilipin-2

## Abstract

**Background/Objectives:** Non-alcoholic fatty liver disease (NAFLD), also referred to as metabolic dysfunction-associated fatty liver disease (MAFLD), is the most common chronic liver disease, closely associated with obesity and Metabolic Syndrome (MetS). Perilipin-2 (PLIN2), the most abundant lipid droplet protein in the liver, is linked to lipid accumulation and inflammation, the hallmarks of NAFLD. The role of PLIN2 in NAFLD etiopathogenesis remains partially understood. This study aims to elucidate the relationship between serum PLIN2 levels and other disease-related parameters in NAFLD, investigate the role of PLIN2 in disease pathogenesis, and evaluate its utility as a biomarker in NAFLD diagnosis. **Methods:** The study included 46 patients diagnosed with NAFLD who presented internal medicine outpatient clinics and 44 healthy controls. **Results:** Serum PLIN2 level was found to be statistically significantly higher in the NAFLD patient group compared to the control group. In the NAFLD group, a statistically significant positive correlation was detected between PLIN2 and Body Mass Index (BMI), hip circumference, C-reactive protein (CRP), and platelet count. In ROC analysis, taking the cut-off value for serum PLIN2 level as 5.52 ng/mL predicted the diagnosis of NAFLD with 50% sensitivity and 97.7% specificity. **Conclusions:** PLIN2 determination demonstrated high specificity at the proposed cut-off value and may represent a promising complementary biomarker for NAFLD, particularly when interpreted alongside other clinical and laboratory parameters. Circulating PLIN2 appears to be influenced by metabolic and inflammatory parameters.

## 1. Introduction

Non-alcoholic fatty liver disease (NAFLD), also referred to as metabolic dysfunction-associated fatty liver disease (MAFLD), is a metabolic disorder characterized by the accumulation of more than 5% fat in hepatocytes, closely associated with other metabolic abnormalities such as Type 2 Diabetes Mellitus (T2D), obesity, hypercholesterolemia, and insulin resistance (IR) [1]. To resolve issues associated with the former NAFLD nomenclature, APASL pioneered the adoption of the MAFLD definition, shifting the diagnostic focus to the presence of metabolic dysfunction as the primary driver of the disease [2,3]. This condition encompasses a spectrum of liver diseases, ranging from simple hepatic steatosis to more severe outcomes like non-alcoholic steatohepatitis (NASH), liver fibrosis, cirrhosis, and ultimately, hepatocellular carcinoma (HCC) [4]. NAFLD is recognized as the most common cause of chronic liver disease [5]. Its prevalence varies depending on factors such as the diagnostic method utilized, age, sex, and ethnicity; however, it is generally accepted to affect approximately one-quarter of the global population [6].

The gold-standard method for the diagnosis of NAFLD patients is liver biopsy. However, ultrasound (USG), computed tomography (CT), and magnetic resonance imaging (MRI) techniques are mostly used for its diagnosis and follow-up. Ultrasonography is the most widely used first-line imaging modality for the detection of hepatic steatosis in clinical practice [7]. European recommendations support the utilization of serum biomarkers and calculated scores as an alternative diagnostic strategy for NAFLD, frequently favoring this approach over traditional screening, particularly in broad epidemiological investigations, even though ultrasound is considered the initial method [7,8].

Lipid Droplets (LDs) are enveloped by a phospholipid monolayer containing specific proteins [9]. Perilipin (PLIN), one of these hallmark proteins, plays a crucial role in LD dynamics [10]. PLIN coating the LD surface inhibits the hydrolysis of cytosolic triglycerides (TG) by hormone-sensitive lipase (HSL). Both PLIN and HSL are activated via phosphorylation by cyclic adenosine monophosphate (cAMP)-dependent protein kinase A (PKA). This phosphorylation step is central to HSL regulation and constitutes the rate-limiting step of lipolysis [11]. Through this mechanism, PLIN mediates the regulation between LDs and the cellular environment. Previous studies have demonstrated that PLIN inhibits lipid catabolism and promotes lipid accumulation. Five distinct subtypes of PLIN exist, exhibiting differential expressions across various tissues. PLIN2,which is also referred to as adipophilin or adipose differentiation-related protein (ADFP), represents the predominant PLIN subtype found in macrophages that are laden with cholesterol [12].

To the best of our knowledge, this is one of the first studies to specifically investigate the significance of serum PLIN2 levels as a potential early indicator of inflammation in patients with NAFLD [13]. This search attempts to fill a gap in the literature regarding the non-invasive diagnostic potential of this specific lipid droplet protein.

The primary objective of this study is to elucidate the connection between serum PLIN2 levels and various disease-related parameters in NAFLD, an inflammatory process. We aim to investigate the potential role of PLIN2 in the etiopathogenesis of NAFLD and, crucially, to assess the feasibility of using circulating PLIN2 levels as a novel biomarker for NAFLD diagnosis.

## 2. Materials and Methods

### 2.1. Study Design

We designed the study as a prospective case–control investigation performed at a single tertiary care center in Istanbul. All participants were recruited from the Internal Medicine outpatient clinics of Kanuni Sultan Suleyman Training and Research Hospital between 1 November 2022, and 1 January 2023. All procedures were carried out in accordance with the Declaration of Helsinki and were approved by the Kanuni Sultan Suleyman Training and Research Hospital ethics committee (Approval No: 2022.10.203; Date: 20 October 2022).

### 2.2. Subjects

The study population consisted of individuals who presented it to the Internal Medicine outpatient clinics of Istanbul Kanuni Sultan Suleyman Training and Research Hospital during the study period.

The case group included adults diagnosed with non-alcoholic fatty liver disease (NAFLD) based on abdominal ultrasonography showing at least grade 2 steatosis. Only those with a Hepatic Steatosis Index (HSI) of 36 or higher were enrolled to ensure diagnostic accuracy. NAFLD diagnosis was established using non-invasive criteria, combining ultrasonographic findings with HSI, after exclusion of secondary causes.

The control group consisted of volunteers with normal abdominal ultrasonographic findings, no known chronic diseases, and no abnormal laboratory results. These participants had no history of alcohol consumption or metabolic comorbidities.

**Figure 1:**
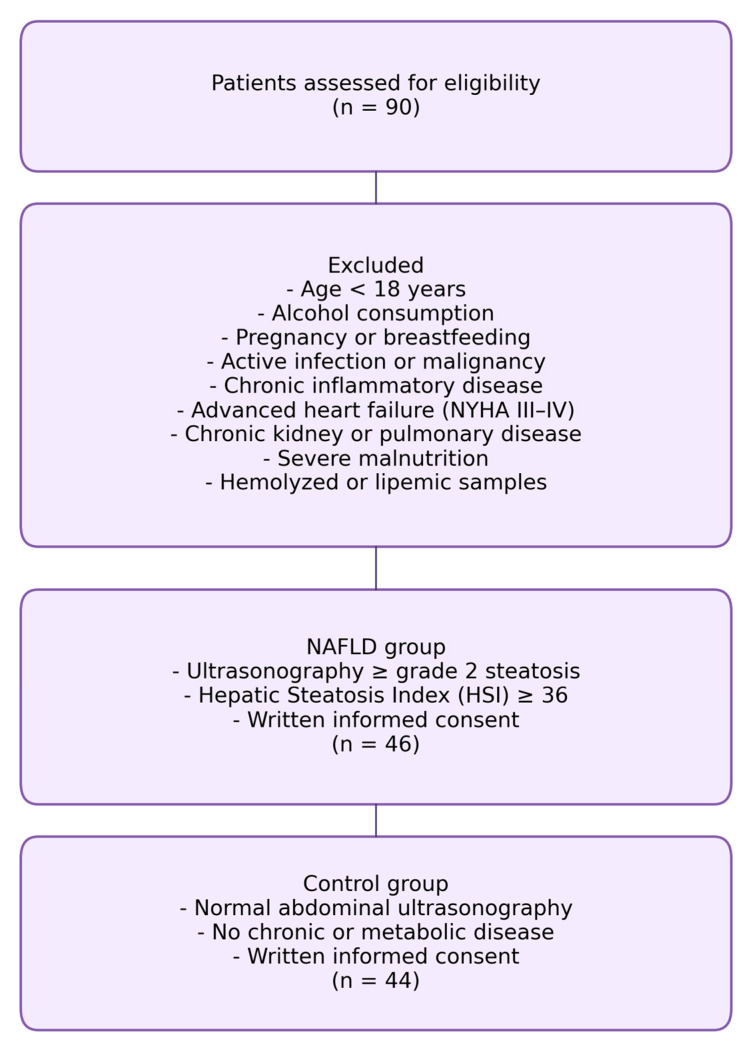
Flowchart illustrates the inclusion and exclusion criteria of the study population.

All individuals agreed to participate after receiving detailed information about the study’s purpose and procedures. The flowchart illustrating the inclusion and exclusion criteria of the study population is presented in Figure 1.

### 2.3. Inclusion Criteria

Participants were eligible for the NAFLD group if they were 18 years of age or older, had no history of alcohol consumption, and were diagnosed with NAFLD. Diagnosis required the presence of at least grade 2 hepatic steatosis on ultrasonography together with an HSI score of ≥36. Only individuals who voluntarily agreed to participate were included.

For the control group, inclusion required being 18 years or older, having no alcohol use, no known chronic or metabolic disease, normal abdominal ultrasonography findings, and normal routine laboratory parameters. Written informed consent was obtained from all volunteers.

### 2.4. Exclusion Criteria

Individuals were excluded if they were younger than 18 years of age, were pregnant or breastfeeding, or reported any alcohol use.

Participants with active infections, malignancy, chronic inflammatory diseases, advanced heart failure (stage III–IV), or severe malnutrition were also not eligible.

Those with chronic kidney disease requiring hemodialysis or peritoneal dialysis, as well as individuals with chronic pulmonary disorders including chronic obstructive pulmonary disease (COPD), bronchiectasis, asthma, or pulmonary hypertension excluded from the study. Hemolyzed or lipemic blood samples were not accepted for biochemical analyses.

### 2.5. Data Collection

Data for the study was gathered during routine outpatient visits. At the initial assessment, each participant’s age, sex, smoking habits, existing medical conditions, and current medications were noted.

Anthropometric measurements were taken by clinic staff in accordance with standard practice. Body weight and height were measured with a SECA 213 device (Hamburg, Germany), and these values were used to calculate BMI. Weight was recorded with the participant wearing light clothing and no shoes. Height was measured with the patient standing upright against the stadiometer.

Waist measurements were taken at the level between the lowest rib and the iliac crest, while hip circumference was recorded at the point of most significant gluteal prominence. These values were later used to calculate the waist-to-hip ratio.

Waist circumference was measured at the midpoint between the lower rib margin and the iliac crest, and hip circumference at the level of maximum gluteal prominence. The waist-to-hip ratio was subsequently calculated.

Blood pressure was measured with an automated device (Omron HEM-7120, Omron Healthcare Co., Ltd., Kyoto, Japan), and the average of three consecutive readings was used as the final value.

Routine laboratory parameters including ALT, AST, fasting glucose, HOMA-IR, HbA1c, lipid profile (LDL-C, HDL-C, triglycerides, total cholesterol), CRP, platelet count, and lymphocyte count were retrieved from the hospital’s electronic medical record system.

Metabolic syndrome was defined according to the 2005 International Diabetes Federation (IDF) criteria, which require central obesity plus at least two additional metabolic risk factors [14,15].

### 2.6. Sample Collection

After an overnight fast of at least eight hours, venous blood samples were obtained from the antecubital vein between 08:00 and 10:00 in the morning. The collected blood was immediately transferred into standard vacuum tubes and centrifuged at 4000 rpm for 10 min.

The serum fraction was separated promptly and stored at −80 °C until biochemical analysis. All samples were thawed only once before testing. Hemolyzed or lipemic specimens were excluded.

### 2.7. Biochemical Analyses

#### Assessment of Serum Perilipin-2 (PLIN 2) Concentration

Serum PLIN2 concentration was determined using a commercially available sandwich Enzyme-Linked Immunosorbent Assay (ELISA) kit (Human ADRP/Perilipin-2, Cat No: E-EL-H0278, elabscience, Biotechnology Inc., Houston, TX, USA), strictly adhering to the manufacturer’s protocol. All samples were examined twice. The calculated serum PLIN2 levels were quantified in ng/mL by interpolating the values from a generated standard curve. Intra- and inter-CV for PLIN2 were determined to be 7.8% and 9.3%, respectively.

### 2.8. Statistical Analysis

All statistical analyses were performed using SPSS for Windows, version 26.0 (IBM Corp., Armonk, NY, USA). The distribution of continuous variables was examined with the Shapiro–Wilk test. Variables with normal distribution were presented as mean ± standard deviation, while non-normally distributed data were expressed as median values (minimum–maximum). Categorical variables were reported as numbers and percentages. Group comparisons were performed using Student’s *t*-test for normally distributed variables and the Mann–Whitney U test for non-parametric variables. Associations between categorical variables were evaluated using the chi-square test. Correlation analyses were conducted using Pearson’s correlation coefficient for normally distributed variables and Spearman’s rank correlation coefficient for those that did not meet normality assumptions. The diagnostic performance of serum PLIN2 levels was assessed using receiver operating characteristic (ROC) curve analysis. A *p*-value < 0.05 was considered statistically significant.

## 3. Results

### 3.1. Baseline Characteristics

A total of 90 participants were included in the study, comprising 46 patients with NAFLD and 44 healthy controls. The two groups were comparable in terms of age, sex distribution, height, and smoking status (*p* > 0.05). Body weight, BMI, waist circumference, hip circumference, and waist-to-hip ratio were all significantly higher in the NAFLD group compared with controls (all *p* < 0.001). Among patients with NAFLD, 41.3% had T2D, 30.4% had hypertension, 67.3% had dyslipidemia, and 67.4% met the diagnostic criteria for MetS (Table 1).

### 3.2. Biochemical Findings

Biochemical data are summarized in Table 2. Patients with NAFLD had significantly higher fasting glucose, HOMA-IR, HbA1c, AST, ALT, triglycerides, CRP, and lymphocyte count than healthy controls (all *p* < 0.05). HDL-C was significantly lower in the NAFLD group, whereas total cholesterol, LDL-C, and platelet counts did not differ significantly between groups (*p* > 0.05).

### 3.3. Serum PLIN2 Levels

**Figure 2:**
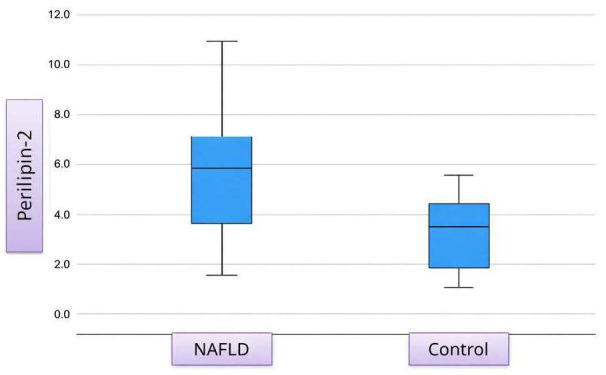
Distribution of Serum Perilipin-2 Levels in the NAFLD and Control Groups.

Serum PLIN2 concentrations were markedly elevated in patients with NAFLD compared with healthy individuals (median [IQR]: 5.66 vs. 3.46 ng/mL; *p* < 0.001) (Table 3, Figure 2).

### 3.4. Correlation Analyses

Correlation analysis revealed that serum PLIN2 levels were positively associated with BMI, hip circumference, CRP, and platelet count in the NAFLD group (all *p* < 0.05). No significant correlations were observed between PLIN2 and other anthropometric or biochemical variables (Table 4).

### 3.5. Subgroup Analyses

Subgroup comparisons showed that PLIN2 levels were significantly higher in women than in men (*p* = 0.003). Smoking status, diabetes, hypertension, dyslipidemia, and MetS did not considerably influence PLIN2 concentrations. Across obesity classes, PLIN2 levels differed significantly, with the main difference observed between class I and class II obesity.

### 3.6. Diagnostic Performance of PLIN2

**Figure 3:**
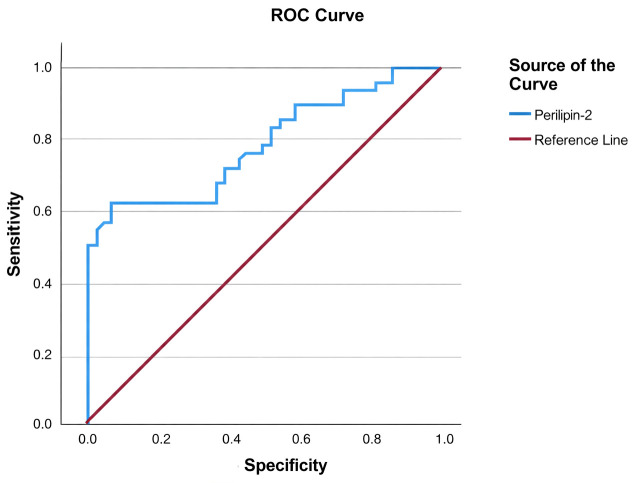
Receiver operating characteristic (ROC) curve showing the diagnostic performance of serum Perilipin-2 for identifying NAFLD.

The diagnostic accuracy of serum PLIN2 for identifying NAFLD was assessed using ROC curve analysis. A cut-off value of 5.52 ng/mL yielded 50% sensitivity and 97.7% specificity, with an AUC of 0.784 (95% CI: 0.689–0.879; *p* < 0.05) (Figure 3, Table 5).

## 4. Discussion

The current study’s most significant finding is the statistically significant elevation of serum PLIN2 levels in NAFLD patients compared to controls, supporting its potential as a novel metabolic biomarker. ROC analysis demonstrated a remarkably high specificity (97.7%) at the proposed cut-off value of 5.52 ng/mL; however, the relatively low sensitivity should be taken into consideration when interpreting the diagnostic performance of serum PLIN2. Accordingly, PLIN2 may be more appropriately regarded as a complementary or confirmatory biomarker rather than an independent diagnostic marker. Furthermore, PLIN2 levels correlated positively with key indicators of adiposity and inflammation, specifically BMI, hip circumference, CRP, and platelet count, confirming its link to the metabolic and inflammatory components of the disease. Notably, PLIN2 levels were significantly higher in female patients and varied significantly across obesity classes, suggesting potential clinical utility in risk stratification. Taken together, these findings suggest that circulating PLIN2 reflects adiposity- and inflammation-related metabolic alterations in NAFLD and may serve as a promising non-invasive biomarker, particularly in combination with other clinical and laboratory parameters.

### 4.1. Obesity, Anthropometrics, and PLIN2

Overweight and obesity constitute major public health concern due to their strong association with a wide spectrum of chronic diseases, including T2D, cardiovascular diseases, and cancer. While BMI remains the most common metric for assessing risk and prevalence, parameters such as waist circumference, hip circumference, and the waist-to-hip ratio are also widely employed for risk assessment [16]. Mendelian randomization studies, such as the one by Yuan et al. [17], have confirmed the roles of lifestyle and metabolic factors including smoking, obesity, high systolic blood pressure, low HDL-C, and high triglycerides (TG) in NAFLD development, supporting a strong link between obesity (beyond BMI alone) and NAFLD. In the current study, the NAFLD patient group exhibited significantly higher values for weight, BMI, waist circumference, hip circumference, and waist-to-hip ratio compared to healthy controls. Furthermore, our study found a significant association between NAFLD status, obesity, and PLIN2. Specifically, a statistically significant positive correlation was observed between serum PLIN2 levels and both BMI and hip circumference in the NAFLD group. These findings are consistent with the comparative study by Conte et al. [13], which also reported a significant correlation between PLIN2 and BMI, thus supporting our results on the interplay between PLIN2, adiposity, and metabolic dysfunction.

### 4.2. NAFLD, IR, and T2D

The liver’s fundamental role in regulating glucose homeostasis and gluconeogenesis suggests a close relationship between NAFLD, T2D, and IR [18]. It is hypothesized that IR and T2D contribute to NAFLD development via associated chronic inflammation and immunological changes [19], a relationship consistently documented across numerous studies [20]. Sporea et al. [21] investigated hepatic steatosis and fibrosis in T2D patients, finding that female gender, high BMI, waist circumference, elevated AST, total cholesterol, TG, and glucose levels were associated with severe steatosis. Furthermore, high BMI, waist circumference, AST, and HbA1c levels were linked to advanced fibrosis, emphasizing that obesity increases the risk for both steatosis and fibrosis development. Consistent with this body of literature, the current study also found statistically significantly higher levels of glucose, HbA1c, and HOMA-IR in the NAFLD patient group compared to the healthy controls. This consistent finding underscores the critical association between NAFLD and impaired glucose metabolism, highlighting that IR is a fundamental component of the disease’s pathogenesis in the studied cohort.

### 4.3. Dyslipidemia and Lipid Metabolism in NAFLD

Dyslipidemia, characterized by impaired blood lipid levels, frequently coexists with NAFLD. Elevated fatty acid levels in both the liver and blood are considered critical factors in NAFLD development [22], with increased TG accumulation in hepatocytes being a central pathophysiological feature [23]. Supporting this, a study by Li et al. [24] investigating the relationship between the Triglyceride-Glucose Index (TyG) and NAFLD in T2D patients found a strong positive association between a higher TyG index and increased NAFLD risk. Furthermore, NAFLD is often reported to be more prevalent in patients presenting with low serum HDL-C, high TG, and elevated BMI, ALT, and AST levels [24]. Consistent with the established literature, the present study similarly found statistically significantly higher serum TG levels and lower serum HDL-C levels in the NAFLD patient group compared to the control group. Dyslipidemia, characterized by impaired blood lipid levels, frequently coexists with NAFLD.

### 4.4. CRP, Inflammation, and the PLIN2 Connection

CRP is a widely utilized, reliable, and cost-effective positive acute-phase protein marker for inflammation, used in the diagnosis and monitoring of numerous diseases [25]. Elevated levels of CRP, IL-1beta, IL-6, and TNF-alpha have been consistently linked to an increased risk of NAFLD [26], with many studies supporting CRP’s utility in NAFLD diagnosis and severity assessment [27]. Consistent with the literature, Kumar et al. [27], who found a significant association between NAFLD and CRP along with anthropometric markers, the current study observed a statistically significant elevation of serum CRP in the NAFLD patient group compared to controls. Crucially, the study also found a significant positive correlation between serum PLIN2 and CRP within the NAFLD group. This correlation is mechanistically supported by animal studies, such as that by Molangiri et al. [28], which demonstrated the simultaneous increase in hepatic PLIN2 levels and pro-inflammatory mediators (IL-6, CRP) during microvesicular steatosis in rats. This simultaneous increase and positive correlation between serum PLIN2 and CRP strongly positions PLIN2 not just as a lipid storage marker, but as a direct indicator reflecting the underlying, chronic low-grade systemic inflammation characteristic of NAFLD pathogenesis.

### 4.5. Hematological Markers, PLIN2, and Inflammation

NAFLD is a chronic inflammatory condition where lymphocytes play a crucial immune role. A Mendelian randomization study by Zhu et al. [28] analyzing 4198 patients linked NAFLD frequency positively with lymphocyte and reticulocyte counts, alongside elevated hemoglobin, neutrophil, and platelet counts, attributing these changes to the inflammatory response. Moreover, platelet count is a necessary component of non-invasive fibrosis scores like APRI, FIB-4, and NAFLD Fibrosis Score [29,30]. In the present study, the NAFLD group showed significantly higher lymphocyte counts than controls. Although platelet count was higher in the NAFLD group, the difference was not statistically significant. Importantly, a significant positive correlation was established between PLIN2 and platelet count within the NAFLD group. As far as the literature search permits, this specific correlation between circulating PLIN2 and platelet count appears to be a novel finding. While elevated platelet count may be secondary to inflammation, further studies with larger cohorts and simultaneous histological sampling are warranted to fully clarify this novel relationship.

### 4.6. Aminotransferases (AST/ALT)

AST and ALT are routinely used to assess liver function. In NAFLD patients, serum aminotransferases (AST and ALT) are often mildly elevated secondary to hepatocyte inflammation; however, normal levels do not exclude the disease [31]. Consistent with the established literature, the present study found statistically significant elevations in both AST and ALT levels in the NAFLD patient group compared to the control group. The observed, albeit mild, increases in both AST and ALT levels in the NAFLD cohort confirm the presence of ongoing hepatocellular injury, which aligns with the inflammatory nature of the disease, even if these markers are not fully diagnostic.

### 4.7. Gender Differences and PLIN2

The precise impact of gender on NAFLD prevalence and progression remains inconclusive, with conflicting evidence in the literature; some studies suggest women are more affected [32], while others report higher prevalence in men [33]. In the present study, no statistically significant difference in NAFLD prevalence was found between male and female participants across the patient and control groups. However, within the NAFLD patient group, female patients exhibited statistically significantly higher serum PLIN2 levels compared to male patients. This finding is consistent with the results reported by Conte et al. [13], who also observed higher serum PLIN2 levels in women than in men. Conte et al. [13] attributed this difference to the generally higher body fat percentage in women and potential PLIN2 production in tissues like the breast, thus supporting the results of this current study. The significantly higher PLIN2 levels found in female NAFLD patients, mirroring existing literature, suggests that serum PLIN2 may reflect gender-specific differences in adipose tissue distribution or fat metabolism, under scoring its potential role as a sex-dependent metabolic biomarker.

### 4.8. Comorbidities, Metabolic Syndrome, and PLIN2

NAFLD is closely linked to comorbidities such as hypertension (HT), T2D, and dyslipidemia, and is widely regarded as a component of MetS [34]. In this cohort, the NAFLD group showed high rates of comorbidity: 30% with HT, 41% with T2D, and 67% with dyslipidemia. Applying the International Diabetes Federation (IDF) 2005 criteria, 67% of the patients were diagnosed with MetS. Furthermore, none of the patients in the NAFLD group were of normal weight based on WHO BMI classification, with the entire group consisting of overweight and obese individuals. Despite the strong clinical association, the presence of specific comorbidities (HT, T2D), and dyslipidemia) did not significantly affect serum PLIN2 levels in the NAFLD patient group. However, a significant difference in PLIN2 levels was found when patients were stratified by obesity class. The lack of effect from established comorbidities, coupled with the observation that the significant difference in obesity stratification was confined only between Class 1 and Class 2 obese individuals, limits definitive interpretation. We therefore propose that future studies with significantly larger sample sizes and broader demographic representation are necessary to precisely clarify the relationship between serum PLIN2 levels and the full spectrum of NAFLD comorbidities.

### 4.9. Central Role of PLIN2 in NAFLD Pathogenesis

PLIN2 is a pivotal regulator of intracellular lipid accumulation and metabolism, functioning in the formation and stability of LDs within the liver and peripheral tissues [35,36]. Immunohisto-chemical evidence confirms PLIN2’s localization on hepatic LD surfaces in fatty liver, suggesting its direct involvement in NAFLD etiopathogenesis [37]. Mechanistically, PLIN2 inhibits the hydrolysis of TG by blocking lipases from accessing neutral lipids, thereby stabilizing LDs [38]. Experimental data demonstrates that PLIN2 overexpression leads to the excessive hepatic TG accumulation characteristic of NAFLD [39], and PLIN2-knockout mice were resistant to diet-induced steatosis and inflammation [40]. Furthermore, PLIN2 expression is stimulated by reactive oxygen species (ROS), driving lipid accumulation. Given that modulating PLIN2 can mitigate steatosis (e.g., octreotide treatment reversing PLIN2 increase in obese rats [39], targeting PLIN2 synthesis represents a potential therapeutic avenue for NAFLD.The significant elevation of serum PLIN2 levels found in this study is strongly supported by robust experimental evidence demonstrating PLIN2’s critical regulatory role in hepatic lipid droplet stability and its contribution to steatosis, reinforcing its potential as a clinically relevant biomarker for NAFLD. Consistent with this robust literature, the current study found statistically significantly elevated serum PLIN2 levels in the NAFLD patient group, supporting the hypothesis that PLIN2 acts as an active and effective biomarker reflecting NAFLD development.Although PLIN2 is primarily an intracellular lipid droplet-associated protein expressed in hepatocytes, several mechanisms may account for its presence in the circulation. Hepatocellular injury, increased lipid droplet turnover, and enhanced membrane permeability in the setting of metabolic stress and inflammation may facilitate the release of PLIN2 into the bloodstream [39,40]. In addition, experimental studies have demonstrated that PLIN2 expression is upregulated under steatotic conditions and metabolic stress, which may further contribute to detectable serum levels [41]. Therefore, circulating PLIN2 may reflect hepatocellular lipid overload and metabolic inflammation rather than direct tissue expression, supporting its biological plausibility as a serum biomarker.

### 4.10. Diagnostic Efficacy of PLIN2 (ROC Analysis)

In the present study, a ROC curve analysis was performed to assess the diagnostic utility of serum PLIN2 levels for NAFLD identification. The optimal cut-off value was determined to be 5.52 ng/mL at which PLIN2 was found to predict NAFLD diagnosis with 50% sensitivity and a remarkably high 97.7% specificity. The high specificity observed at the identified cut-off suggests that serum PLIN2 may be useful for confirming the presence of NAFLD in selected clinical contexts, although its moderate sensitivity limits its broader diagnostic applicability when used alone.

### 4.11. The PLIN2–Lysosomal Acid Lipase (LIPA) Axis: Mechanistic Link to Metabolic Dysfunction

Our finding of elevated serum PLIN2 levels in NAFLD patients aligns with recent mechanistic evidence, particularly Fang et al. [41], who showed that exercise ameliorates hepatic lipid accumulation in a mouse NAFLD model by modulating the PLIN2-LIPA axis, a key regulator of lipophagy. In this pathway, PLIN2 must be properly regulated to enable LD breakdown via LIPA-mediated autophagy. Fang et al. [41] demonstrated that disruption of this interaction impairs lipid clearance, highlighting PLIN2’s active role in lipid metabolism rather than serving merely as a fat storage marker. Consistently, our observation of elevated circulating PLIN2 alongside increased BMI and CRP suggests a dysfunction of this PLIN2-LIPA dependent lipophagy mechanism in NAFLD. Thus, serum PLIN2 may reflect not only hepatic lipid burden but also a specific failure in lipid clearance, reinforcing its potential as a biomarker of metabolic dysregulation in NAFLD.

### 4.12. The Limitations of This Study

Despite providing significant insights into the association between serum PLIN2 levels and NAFLD, several limitations warrant consideration. Foremost, the cross-sectional, single-center design precludes the establishment of causal relationships and limits the generalizability of the findings, necessitating prospective, longitudinal validation in larger multicenter cohorts to define the prognostic value of PLIN2 in disease onset. Furthermore, the reliance on non-invasive diagnostic tools—specifically transabdominal ultrasonography and laboratory parameters—rather than the histopathological gold standard of liver biopsy may have resulted in decreased sensitivity for detecting mild steatosis (<20–30%) and an inability to accurately differentiate between simple steatosis and non-alcoholic steatohepatitis (NASH). This lack of histopathological staging or advanced elastography-based grading further constrained the evaluation of PLIN2’s correlation with the severity of hepatic inflammation or fibrosis. Additionally, the modest sample size may have restricted the statistical power required to robustly define and validate ROC-derived cut-off values, suggesting that the reported diagnostic metrics remain preliminary. Finally, despite adjusting for conventional metabolic variables, unmeasured confounders such as specific dietary patterns, physical activity levels, and genetic polymorphisms (e.g., *PNPLA3* or *TM6SF2* variants) known to modulate lipid droplet dynamics may have influenced serum PLIN2 concentrations. Given that PLIN2 is expressed in various non-adipose tissues, the potential contribution of subclinical extra-hepatic comorbidities to systemic levels cannot be entirely excluded, highlighting the need for further investigation into the pathophysiological specificity of PLIN2 within the heterogeneous NAFLD spectrum.

### 4.13. Clinical Implications

The present findings indicate that serum PLIN2 exhibits a high specificity at the proposed cut-off value, suggesting potential utility as a non-invasive biomarker in selected clinical contexts. However, given its limited sensitivity and the observed associations with metabolic and inflammatory markers such as BMI and CRP, circulating PLIN2 may reflect systemic metabolic inflammation rather than liver-specific pathology. Accordingly, PLIN2 should be interpreted with caution and considered as a complementary biomarker rather than a primary diagnostic indicator. Additional analyses incorporating multivariate adjustment and formal disease staging would help clarify whether serum PLIN2 is independently associated with NAFLD.

## 5. Conclusions

Serum PLIN2 levels were significantly elevated in patients with NAFLD compared to healthy controls and were associated with key metabolic parameters and the inflammatory marker CRP. At the proposed cut-off value of 5.52 ng/mL, serum PLIN2 showed a high specificity (97.7%). Nevertheless, the relatively limited sensitivity suggests that serum PLIN2 may be better viewed as a complementary or confirmatory biomarker rather than an independent diagnostic tool. The associations observed with anthropometric measures and inflammatory markers indicate that circulating PLIN2 reflects underlying metabolic and inflammatory alterations related to NAFLD. Further large-scale, longitudinal studies with histological confirmation are needed to validate these findings across different stages of disease.

## Figures and Tables

**Table 1:** Comparison of Baseline Demographic and Anthropometric Variables Between Groups.

Variables	Control Group (*n* = 44)	NAFLD Group (*n* = 46)	*p*
Age (years)	43.70 ± 6.04 [43]	44.3 ± 4.71 [45]	0.060 *^1^
Sex			
Male	21 (47.73%)	22 (47.83%)	0.993 *^3^
Female	23 (52.27%)	24 (52.17%)	
Smoking	18 (49.91%)	23 (50.00%)	0.513 *^3^
Height (m)	1.68 ± 0.09 [1.66]	1.64 ± 0.10 [1.61]	0.055 *^1^
Weight (kg)	69.34 ± 18.83 [68.5]	93.1 ± 16.56 [90,95]	<0.001 *^1^
BMI (kg/m^2^)	24.51 ± 3.72 [24.32]	34.81 ± 5.57 [33.94]	<0.001 *^1^
Waist circumference (cm)	84.59 ± 13.01 [87]	112.98 ± 10.91 [111.5]	<0.001 *^1^
Hip circumference (cm)	97.70 ± 11.36 [96.5]	117.74 ± 9.84 [116]	<0.001 *^1^
Waist-to-hip ratio	0.86 ± 0.06	0.96 ± 0.07	<0.001 *^2^

Data are presented as mean ± standard deviation or *n* (%) [median]. NAFLD: Non-alcoholic fatty liver disease. *p*-values were calculated using the Mann–Whitney U test (*^1^), Student’s *t*-test (*^2^), or chi-square test (*^3^). A *p*-value < 0.05 was considered statistically significant.

**Table 2:** Biochemical Parameters of Study Population.

Variables	Control Group (*n* = 44)	NAFLD Group (*n* = 46)	*p*
Glucose (mg/dL)	92.23 ± 9.15 [91.5]	121.83 ± 54.05 [99]	0.002 *^1^
HOMA-IR	2.43 ± 1.84 [2.05]	7.23 ± 9.11 [4.13]	<0.001 *^1^
HbA1c (%)	5.47 ± 0.36 [5.4]	6.46 ± 1.39 [6.1]	<0.001 *^1^
ALT (U/L)	17.16 ± 11.61 [15]	31.98 ± 17.34 [25.5]	<0.001 *^1^
AST (U/L)	16.93 ± 5.30 [15]	24.20 ± 11.19 [21]	<0.001 *^1^
LDL-C (mg/dL)	108.20 ± 30.12	119.61 ± 37.34	0.114 *^2^
HDL-C (mg/dL)	53.36 ± 16.00 [50.5]	44.15 ± 10.20 [44.5]	0.003 *^1^
Triglycerides (mg/dL)	121.98 ± 70.86 [108]	181.15 ± 110.75 [156.5]	0.001 *^1^
Total cholesterol (mg/dL)	186.55 ± 34.13	197.98 ± 42.52	0.164 *^2^
CRP (mg/L)	2.62 ± 4.63 [1.41]	4.72 ± 4.71 [2.7]	<0.001 *^1^
Platelet count (×10^3^/µL)	257.41 ± 59.88 [245]	283.89 ± 91.72 [272]	0.092 *^1^
Lymphocyte count (×10^3^/µL)	2.21 ± 0.55 [2.1]	2.67 ± 0.70 [2.55]	0.001 *^1^

Data are presented as mean ± standard deviation or median [IQR]. NAFLD: Non-alcoholic fatty liver disease; HOMA-IR: Homeostasis Model Assessment of Insulin Resistance; ALT: Alanine aminotransferase; AST: Aspartate aminotransferase; CRP: C-reactive protein. *p*-values were calculated using the Mann–Whitney U test (*^1^) or Student’s t-test (*^2^). A *p*-value < 0.05 was considered statistically significant.

**Table 3:** Serum PLIN2 Levels in NAFLD Patients and Healthy Controls.

Variable	Control Group (*n* = 44)	NAFLD Group (*n* = 46)	*p*
PLIN2 (ng/mL)	3.23 ± 1.40 [3.46]	5.45 ± 2.26 [5.66]	<0.001

Data are presented as mean ± standard deviation and median [IQR]. *p*-values were calculated using the Mann–Whitney U test. NAFLD: Non-alcoholic fatty liver disease; PLIN2: Perilipin-2.

**Table 4:** Correlation Between Serum PLIN2 Levels and Clinical/Biochemical Parameters in the NAFLD Group.

Variables	r	*p*
Age (years)	0.171	0.107
Height (m)	−0.282	0.058
Weight (kg)	0.063	0.679
BMI (kg/m^2^)	0.431	0.003 (*p* < 0.01)
Waist circumference (cm)	0.120	0.428
Hip circumference (cm)	0.350	0.017 (*p* < 0.05)
Waist-to-hip ratio	−0.216	0.149
Glucose (mg/dL)	−0.090	0.550
HOMA-IR	0.086	0.569
HbA1c (%)	−0.098	0.518
ALT (U/L)	−0.205	0.172
AST (U/L)	−0.133	0.379
LDL-C (mg/dL)	−0.275	0.064
HDL-C (mg/dL)	0.125	0.408
Triglycerides (mg/dL)	−0.107	0.480
Total cholesterol (mg/dL)	−0.194	0.196
CRP (mg/L)	0.337	0.022 (*p* < 0.05)
Platelet count (×10^3^/µL)	0.359	0.014 (*p* < 0.05)
Lymphocyte count (×10^3^/µL)	0.126	0.405

Correlation was assessed using Spearman’s correlation test. A *p*-value < 0.05 was considered statistically significant. BMI: Body mass index; HOMA-IR: Homeostasis model assessment of insulin resistance; ALT: Alanine aminotransferase; AST: Aspartate aminotransferase; CRP: C-reactive protein.

**Table 5:** Diagnostic Performance of Serum PLIN2 for NAFLD Based on ROC Analysis.

NAFLD	AUC (95% CI)	Cut-Off	Sensitivity (%)	Specificity (%)	*p*
Perilipin-2	0.784 (0.689–0.879)	5.52	50	97.77	<0.05

NAFLD: Non-alcoholic fatty liver disease; CI: Confidence interval.

## Data Availability

The data generated and analyzed during this study are available from the corresponding authors upon reasonable request.
